# Risk of Self-Reported Penicillin Allergy Despite Removal of Penicillin Allergy Label: Secondary Analysis of the PALACE Randomized Clinical Trial

**DOI:** 10.1001/jamanetworkopen.2024.29621

**Published:** 2024-08-15

**Authors:** Ana Maria Copaescu, Sara Vogrin, Abby Douglas, Nicholas A. Turner, Elizabeth J. Phillips, Natasha E. Holmes, Jason A. Trubiano

**Affiliations:** 1 The Research Institute of the McGill University Health Centre, McGill University, McGill University Health Centre (MUHC), Montreal, Quebec, Canada; 2 Department of Infectious Diseases, University of Melbourne, at the Peter Doherty Institute for Infection and Immunity, Victoria, Australia; 3 Department of Medicine, St Vincent’s Hospital, University of Melbourne, Fitzroy, Australia; 4 Department of Infectious Diseases, Peter MacCallum Cancer Centre, Melbourne, Victoria, Australia; 5 Department of Infectious Diseases, Duke University Medical Center, Durham, North Carolina; 6 Center for Drug Safety and Immunology, Vanderbilt University Medical Centre, Nashville, Tennessee

## Abstract

This secondary analysis of adult patients in the Penicillin Allergy Clinical Decision Rule (PALACE) Study investigates the risk of self-reported penicillin allergy despite removal of penicillin allergy label.

## Introduction

Fewer than 5% of penicillin allergies are confirmed on formal testing. However, following a negative test result, patients can fail to have their penicillin allergy label removed or may have the label reinstated erroneously or due to a new reaction. Few studies focus on the long-term follow-up and risk factors associated with the persistence or reinstatement of self-reported penicillin allergy (SRPA) following a successful oral challenge with penicillin and allergy label removal.

## Methods

The Penicillin Allergy Clinical Decision Rule (PALACE) study was the first international, multicenter randomized clinical trial to find that direct oral penicillin challenge (DOC; intervention group) was noninferior to the standard of care of penicillin skin testing followed by an oral challenge (control group)^1^ in adult patients with low-risk penicillin allergy (PEN-FAST score <3).^2^ The protocol of the PALACE study was approved by an institutional review board at Austin Health and subsequently by the independent institutional review board at each site. The trial was conducted using the CONSORT reporting guideline. A 6-month postrandomization scripted telephone questionnaire was conducted to assess SRPA, adverse events (AEs), and factors associated with SRPA.^3^ Univariable and multivariable logistic regression was used for analysis; 2-sided *P* < .05 was considered statistically significant. Additional details about the PALACE study can be found in the study protocol (Supplement 1) and statistical analysis plan (Supplement 2). See eMethods in Supplement 3 for additional methods.

## Results

Among 377 patients delabeled in the initial study (247 [65.5%] female; 350 [92.8%] White), 351 (93.1%) completed the 6-month follow-up, and the rest were not reachable by telephone. Of these 351 patients, 23 (6.6%) described SRPA (intervention group: 13 of 178 [7.3%]; control group: 10 of 173 [5.8%]) and 34 (9.7%) reported avoiding penicillin at 6 months (Tables 1 and 2). Of the 23 with SRPA, 11 (47.8%) reported an AE during the 5-day study period, 7 (30.4%) were associated with a new AE, and 5 (21.7%) identified as having a SRPA without an AE (Table 1). Among the AEs reported after the 5-day initial follow-up and associated with new penicillin-based antibiotic treatment, 11 of 13 were immune-mediated (11 of 351 [0.3%]).

**Table 1.** Adverse Events, Phenotypes, and Patient Characteristics of Patients With or Without SRPA^a^

Item	No. (%)		OR (95% CI)	*P* value
	Absence of SRPA (N = 328)	SRPA (N = 23)		
Randomization group				
Intervention group	165 (50.3)	13 (56.0)	1 [Reference]	NA
Control group	163 (49.7)	10 (44.0)	0.78 (0.33-1.83)	.57
Demographic characteristics				
Age, median (IQR), y	52 (36-66)	56 (40-67)	1.01 (0.98-1.04)	.43
Sex				
Female	217 (66.2)	15 (65.2)	1 [Reference]	NA
Male	111 (33.8)	8 (34.8)	1.04 (0.43-2.53)	.93
Race				
White	305 (93.0)	21 (91.3)	1 [Reference]	NA
Other^b^	23 (7.0)	2 (8.7)	1.26 (0.28-5.72)	.76
Recruiting site				
Australia	166 (50.6)	9 (39.1)	1 [Reference]	NA
Canada	118 (36.0)	9 (39.1)	1.41 (0.54-3.65)	.48
US	44 (13.4)	5 (21.7)	2.10 (0.67-6.57)	.20
PEN-FAST score				
0	133 (40.5)	8 (34.8)	1 [Reference]	NA
1	179 (54.6)	15 (65.2)	1.39 (0.57-3.38)	.46
2	16 (4.9)	0	NA	NA
Timing of the index reaction				
<5 y	6 (1.8)	0	1 [Reference]	NA
Immediate reaction (<2 h after dose)	36 (11.0)	4 (17.4)	1.71 (0.55-5.30)	.35
Childhood reaction	201 (61.3)	10 (43.5)	0.49 (0.21-1.14)	.10
Adverse events				
Adverse events within 2 d				
Any	20 (6.1)	10 (43.5)	11.85 (4.63-30.34)	<.001
Immune-mediated	6 (1.8)	3 (13.0)	8.05 (1.87-34.58)	.005
Non–immune-mediated	15 (4.6)	7 (30.4)	9.13 (3.26-25.53)	<.001
Adverse events 2-5 d				
Any	4 (1.2)	1 (4.3)	3.68 (0.39-34.36)	.25
Immune-mediated	3 (0.9)	1 (4.3)	4.92 (0.49-49.31)	.18
Non–immune-mediated	1 (0.3)	0	NA	
Adverse events after 5 d				
Any	6 (1.8)	7 (30.4)	23.48 (7.07-77.99)	<.001
Immune-mediated	4 (1.2)	7 (30.4)	35.44 (9.40-133.60)	<.001
Non–immune-mediated	2 (0.6)	0	NA	
No AE at any time	299 (91.2)	5 (21.7)	0.03 (0.01-0.08)	<.001
Antibiotics received subsequent to delabeling				
Any antibiotic	123 (37.5)	17 (73.9)	4.72 (1.81-12.30)	.001
Penicillins^c^	66 (20.1)	10 (43.5)	3.05 (1.28-7.27)	.01
Cephalosporins^d^	18 (5.5)	2 (8.7)	1.64 (0.36-7.55)	.53
Other^e^	21 (6.4)	5 (21.7)	4.06 (1.37-12.02)	.01
Unknown	21 (6.4)	1 (4.3)	0.66 (0.09-5.17)	.70

Abbreviations: AE, adverse event; NA, not applicable; OR, odds ratio; SRPA, self-reported penicillin allergy.

^a^ Patients with positive skin testing or positive oral challenges were excluded from this analysis.

^b^ The other race category included East Asian (n = 7), Indo Asian (n = 4), African (n = 6), Hispanic or Latino (n = 4), other (n = 4; 1 in each of: Middle Eastern, not reported, Turkish and Hispanic, Afghanistan).

^c^ The penicillins category includes amoxicillin, nafcillin, and penicillin unspecified.

^d^ The cephalosporin category includes cefazolin, cefalexin, cefdinir, cefadroxil, and cefuroxime.

^e^ The other antibiotic category includes ciprofloxacin, clindamycin, trimethoprim/sulfamethoxazole, doxycycline, levofloxacin, azithromycin, roxithromycin, erythromycin, vancomycin, and minocycline.

**Table 2.** Six-Month Postrandomization Telephone Questionnaire Results

Responses to survey questions	No. (%)		*P* value
	Intervention group (N = 178), direct oral challenge	Control group (N = 173), skin testing + oral challenge	
Confirmed having had penicillin testing	177 (99.4)	173 (100.0)	>.99
Reported receiving a delabeling letter^a^	143 (80.3)	141 (81.5)	NA
Safety during testing: I felt safe during the test dose			
Strongly agree	121 (68.0)	129 (74.6)	.10
Agree	54 (30.3)	40 (23.1)	
Neutral	1 (0.6)	4 (2.3)	
Disagree	2 (1.1)	0	
Recommendation testing: I recommend the penicillin assessment to other patients with a penicillin allergy			
Strongly agree	129 (72.5)	129 (74.6)	.54
Agree	44 (24.7)	40 (23.1)	
Neutral	2 (1.1)	4 (2.3)	
Disagree	2 (1.1)	0	
Strongly disagree	1 (0.6)	0	
Results testing: What was the result of your penicillin assessment in the clinic?			
Penicillin allergy removed	173 (97.2)	165 (95.4)	.62
Penicillin allergy confirmed	2 (1.1)	1 (0.6)	
I do not know	3 (1.7)	5 (2.9)	
Antibiotics received			
Any antibiotic	76 (42.7)	64 (37.0)	.28
Penicillins^b^	42 (23.6)	36 (20.8)	
Cephalosporins^c^	9 (5.1)	14 (8.1)	
Other^d^	12 (6.7)	16 (9.2)	
Unknown	12 (6.7)	10 (5.8)	.83
Education			
Answered yes to the question, “Do you feel you know more about penicillin allergies?”	144 (80.9)	149 (86.1)	.25
Answered yes to the question, “Do you feel you know more about your reactions to penicillin?”	157 (88.2)	162 (93.6)	.13
Posttesting penicillin allergy label			
Answered yes to the question, “Are you still avoiding penicillin(s)?”	20 (11.2)	14 (8.1)	.37
Answered yes to the question, “Do you consider yourself allergic to penicillin?”	13 (7.3)	10 (5.8)	.67

Abbreviation: NA, not applicable.

^a^ As per protocol, the delabelling letter was systematically given to the patients after a negative penicillin oral challenge.

^b^ The penicillins category includes amoxicillin, penicillins, and nafcillin.

^c^ The cephalosporin category includes cefazolin, cefalexin, cefdinir, cefadroxil, and cefuroxime.

^d^ The other antibiotic category includes ciprofloxacin, clindamycin, TMP/SMX, doxycycline, levofloxacin, azithromycin, roxithromycin, erythromycin, vancomycin, and minocycline.

SRPA at 6 months was associated with immune-mediated AEs reported after the 5-day initial study period (odds ratio, 35.44 [95.0% CI, 9.40-133.60]; *P* < .001). No baseline characteristics were associated with SRPA.

Among the 23 patients with SRPA, 17 (73.9%) took an antibiotic within 6 months of delabeling (64.7% [11 of 17] penicillin class). In the non-SRPA group, 123 of 328 (37.5%) were prescribed an antibiotic course; 66 of 123 (53.6%) were penicillin (Table 1).

## Discussion

This secondary analysis found that DOC was effective for removing a penicillin allergy label, and this delabeling persisted at 6 months in most patients. A significant proportion successfully received penicillin after delabeling, similar to previous reports.^4^

A small number of patients described a new AE, with the majority of AEs reported in the initial 5-day trial follow-up. The delayed immune-mediated reactions, either by day 5 or 6-month follow-up, were not severe. Thirteen of 328 (3.9%) had a documented immune-mediated AE but did not consider themselves penicillin allergic at follow-up.

Among the AEs reported after the 5-day follow-up and associated with new penicillin-based antibiotic treatment, 11 of 13 were immune-mediated (11 of 351; 0.3%). In a large retrospective study, 11 of 650 patients (1.7%) initially delabeled reported a mild delayed reaction.^5^ A resensitization process is suspected, in the context of repeated exposure.^6^ The fact that 39.8% of our cohort had received an antibiotic course at the 6-month follow-up and penicillin was administered in 54.2% of cases, underlines the importance of the delabeling procedure in our population and the absence of secondary events.

There are some limitations to this study. A medical record review was not performed to directly document the allergy labels. Furthermore, despite successful delabeling and the absence of AEs, some patients were still reluctant to take penicillin. The reasons for this were not investigated, and qualitative assessments of those still avoiding penicillin following negative test results and delabeling are required. Nevertheless, the PALACE study has found that single-dose DOC in low-risk patients reporting a penicillin allergy is safe and effective at immediate (5 days) and prolonged follow-up (6 months).

## Notes

Supplement 1.
Trial Protocol

Supplement 2.
Statistical Analysis Plan

Supplement 3.
eMethods.

Supplement 4.
Data Sharing Statement
