# Supplementary material for: Risk of Self-Reported Penicillin Allergy Despite Removal of Penicillin Allergy Label: Secondary Analysis of the PALACE Randomized Clinical Trial
Source: JAMA Netw Open. 2024 Aug 15;7(8):e2429621. doi: 10.1001/jamanetworkopen.2024.29621 (PMC11327879; doi:10.1001/jamanetworkopen.2024.29621)
Supplement: Supplement 1. — Trial Protocol [file jamanetwopen-e2429621-s001.pdf]

PROTOCOL

The use of penicillin allergy clinical decision rule to enable direct oral penicillin provocation - A multicenter randomized control trial - **PALACE Study**

Protocol Number: PALACE
Version: #4
Date: 09/03/2021

Author/s:
A/Prof Jason Trubiano
Dr Ana Copaescu

Sponsor/s:
Austin Health

CONFIDENTIAL

This document is confidential and the property of Austin Health. No part of it may be transmitted, reproduced, published, or used without prior written authorization from the institution.

Statement of Compliance

This document is a protocol for a research project. This study will be conducted in compliance with all stipulation of this protocol, the conditions of the ethics committee approval, the NHMRC National Statement on ethical Conduct in Human Research (2007) and the Note for Guidance on Good Clinical Practice (CPMP/ICH-135/95).

Table of Contents

Contents

Table of Contents 2

1. **Glossary of Abbreviations & Terms** 7
2. **Study Sites** 8
   a. Study Location/s 8
3. **Introduction/Background Information** 9
   b. Lay Summary 9
   c. Introduction 9
   d. Background information 9
4. **Study Objectives** 10
   e. Hypothesis 10
   f. Study Aims 10
   g. Outcome Measures 10

5. Study Design 11
   h. Study Type & Design & Schedule 11

Figure 1 – Overview of the study design 13

Figure 2 – PEN-FAST clinical decision rule 13

   i. Standard Care and Additional to Standard Care Procedures 14

j. Randomization 14

k. Study methodology 14

6. **Study Population** 14
   l. Recruitment Procedure 14
   m. Inclusion Criteria 15
   n. Exclusion Criteria 15
   o. Consent 15

7. Participant Safety and Withdrawal 15
p. Risk Management and Safety 15
q. Handling of Withdrawals 16
r. Replacements 16
8. Statistical Methods 16
s. Sample Size Estimation & Justification 16
t. Statistical Methods To Be Undertaken 16
9. Storage of Blood and Tissue Samples 17
u. Details of where samples will be stored, and the type of consent for future use of samples 17
10. Data Security & Handling 17
v. Details of where records will be kept & How long will they be stored 17
w. Confidentiality and Security 17
x. Ancillary data 17
11. Appendix 1 – Drug allergy testing concentrations 18
12. Appendix 2 – PEN-FAST Clinical decision rule 19
13. Appendix 3 – Pre-Questionnaire Drug Hypersensitivity 20
14. Appendix 5 – 6 Months follow-up telephone questionnaire - Telephone survey script [11] 22
15. References 24

STUDY SYNOPSIS

Title:	The use of **P**enicillin **A**llergy c**L**inic**A**l de**C**ision rul**E** to enable direct oral penicillin challenge – A multicenter non-inferiority randomized controlled trial – PALACE Study
Short Title:	PALACE Study
Design:	Multi-center non-inferiority randomized clinical trial
Study Centers:	Austin Health<br><br>Peter MacCallum Cancer Center<br><br>Royal Melbourne Hospital<br><br>Centre hospitalier de l'Université de Montréal (CHUM)<br><br>McGill University Health Centre (MUHC)<br><br>Vanderbilt University Medical Center<br><br>Duke University Medical Center
Hospital:	Austin Hospital<br><br>Peter MacCallum Cancer Center<br><br>Royal Melbourne Hospital<br><br>Centre hospitalier de l'Université de Montréal (CHUM)<br><br>McGill University Health Centre (MUHC)<br><br>Vanderbilt University Medical Center<br><br>Duke University Medical Center
Study Question:	Is skin testing required for low risk penicillin allergies with PEN-FAST score of <3?
Intervention	Direct oral challenge in patients with PEN-Fast less than 3
Control	Standard of care: skin testing and, if negative, oral challenge.
Study Objectives:	Evaluate the non-inferiority of direct oral challenge without prior skin testing to standard care (skin testing followed by oral challenge if negative) in low risk patients (determined as PEN-FAST < 3).
Primary outcome	The difference in the proportion of positive oral challenges (i.e. immune-mediated reaction)
Secondary outcomes	**Feasibility outcome measure**:<br>• Proportion of patients referred to the outpatient allergy clinic that are eligible for intervention (i.e randomization) as per protocol [Eligibility to screened ratio]

	• Feasibility of recruitment defined as the proportion of patients consenting to participate in the study as per protocol from eligible patients. [Recruitment to eligibility ratio].<br>• Feasibility of intervention delivery defined as the proportion of patients randomized to the intervention arm who had the intervention delivered as per protocol. [Intervention to recruitment ratio]<br><br>**Safety outcome measures:**<br>• The proportion of patients with a penicillin allergy who experience an antibiotic associated immune mediated adverse event OR severe adverse drug reaction as per protocol definitions<br>• The proportion of patients with a penicillin allergy who experience an antibiotic associated non-immune mediated adverse event<br>• Protocol compliance<br><br>**Exploratory efficacy outcomes**<br>• Proportion of patient with positive Penicillin Skin Testing<br>• Proportion of patients with non-immune mediated positive oral challenges<br>• Proportion of patients with severe adverse reaction – anaphylaxis/death<br>• Time from randomization to delabelling<br>• Number of appointments required for Penicillin delabelling<br>• Assessment with the Pre-Questionnaire and the 6 months follow-up Questionnaire
Inclusion Criteria:	1. Adult patients referred to the outpatient allergy clinic for a penicillin allergy history;<br>2. Willing and able to give consent.
Exclusion Criteria:	1. Patient age is < 18 years;<br>2. Patients with a PEN-FAST score ≥3<br>3. Pregnancy;<br>4. Any other illness that, in the investigator's judgement, will substantially increase the risk associated with subject's participation in this study;<br>5. Patients with history of type A adverse drug reaction, drug-associated anaphylaxis, idiopathic urticaria/anaphylaxis, mastocytosis, serum sickness, blistering skin eruption or acute interstitial nephritis;<br>6. Patients where the allergy history was not able to be confirmed with patient;<br>7. Patients on concurrent antihistamine therapy;<br>8. Patients receiving more than stress dose steroids (i.e. > 50mg QID hydrocortisone [or steroid equivalent]).
Number of Planned Subjects:	380
Investigational product:	Not applicable
Safety considerations:	Serious adverse event as per definition<br>Antibiotic associated immune mediated adverse event as per

	definition<br><br>An independent data safety management board (DSMB) will be established to review the progress of the study and monitor adherence to the protocol, participant recruitment, outcomes, complications, and other issues related to participant safety.
Statistical Methods:	Projected sample size: Assuming 80% power, 5% significance (one-sided) and 5% difference between groups regarded as non-inferiority, the estimated sample size is n=380 (190 per group).<br><br>Primary result will be presented as the absolute difference of the primary outcome between groups with 90% confidence interval. If the upper level of this interval is greater than non-inferiority margin (5%), the null hypothesis cannot be rejected. All other secondary outcomes will be presented as difference in the proportion with 95% CI and OR with 95 % CI. Continuous outcomes will be compared using t-test or rank sum test.
Subgroups:	1. Clinical Immunophenotypes (ex. immediate and delayed reactions)<br>2. Patients with multiple drug allergies versus patients with single drug allergies<br>3. Skin testing for minor vs major determinants<br>4. Skin testing with major determinants diater PPL vs Pre-Pen<br>5. Patient immunosuppressed vs non-immunosuppressed

1. Glossary of Abbreviations & Terms

Abbreviation	Description (using lay language)
ST	Skin testing to asses for drug allergy
PT	Prick Skin testing to asses for drug allergy
IDT	Intra-dermal Skin testing to asses for drug allergy
Oral provocation (challenge)	The provision of a test dose of drug to prove or disprove allergy
Oral penicillin provocation (challenge)	In brief, following informed consent, a dose of penicillin (orally) is given to patients with a 1-2 hours observation period post dose. Vital signs are monitored at baseline and as needed. Emergency medication is available on site (including adrenalin administration).
Negative oral penicillin provocation (challenge)	No antibiotic associated immune mediated reactions.
Serious adverse event	A serious adverse event will be defined as any adverse drug event/experience occurring at any dose that in the opinion of the investigators is causal for any of these outcomes: (1) death; (2) life threatening reaction; (3) inpatient hospitalization; (4) results in persistent or significant disability/incapacity; (5) congenital anomaly or birth defect; or (6) requires intervention to prevent permanent impairment or damage.
Antibiotic Associated Immune Mediated Adverse Event	Any immune mediated [immediate (IgE) or non-immediate (T-cell)] reaction within 48 hours of oral provocations judged by two independent reviewers.
Antibiotic Associated non-immune Adverse Event	An antibiotic associated adverse event will include any non-immune mediated reaction (e.g. nausea, vomiting, diarrhea etc.) within 48 hours of oral provocations judged by two independent reviewers.
Penicillin allergy label	A patient reporting an allergy to any of: penicillin “unspecified”, penicillin VK, penicillin G, amoxicillin, ampicillin, flucloxacillin, dicloxacillin.
Low risk penicillin allergy	Unknown > 10 years, maculopapular rash (MPE) greater than 10 years prior, Type A adverse drug reaction (ADR) as per published definition [1], local injection site reaction, childhood benign exanthema.
Delabelled	The removal of a patient’s reported allergy if no adverse event is noted following direct oral provocation or challenge with implicated drug
NSP	Narrow spectrum penicillin including Penicillin VK, penicillin G, amoxicillin, ampicillin, flucloxacillin, dicloxacillin.
NSB	Narrow spectrum beta-lactam including the NSP + cefazolin and cephalexin.
Restricted antimicrobial agents	Antimicrobial agents that include cefepime, ceftazidime, ceftriaxone, ciprofloxacin, clindamycin, meropenem, moxifloxacin, piperacillin/tazobactam, teicoplanin, tobramycin and vancomycin.
PEN-FAST	Penicillin allergy decision rule

2. Study Sites

a. Study Location/s

Site	Address	Contact Person	Phone	Email
Austin Health	145 Studley Road, Heidelberg VIC 3084	A/Prof Jason Trubiano<br>Ana Copaescu	(03) 94966709<br>0466067000	Jason.trubiano@austin.org.au<br>Ana.copaescu@austin.org.au
Peter MacCallum Cancer Center	305 Grattan Street, Melbourne, VIC 3000	A/Prof Jason Trubiano<br>Ana Copaescu	(03) 94966709<br>0466067000	Jason.trubiano@austin.org.au<br>Ana.copaescu@austin.org.au
Royal Melbourne Hospital	300 Grattan Street, Melbourne, VIC 3000	Joseph De Luca<br>Ana Copaescu	(03) 93493199<br>0466067000	Joseph.DeLuca@mh.org.au<br>Ana.copaescu@austin.org.au
Centre hospitalier de l'Université de Montréal (CHUM)	1051, rue Sanguinet Montréal Québec H2X 3E4, Canada	A/Prof Philippe Bégin<br>Ana Copaescu	0466067000	philippe.begin@umontreal.ca<br>Ana.copaescu@austin.org.au
McGill University Health Centre (MUHC)	1650 Cedar Ave, Montreal, Quebec H3G 1A4, Canada	A/Prof Christos Tsoukas<br>Ana Copaescu	0466067000	christos.tsoukas@mcgill.ca<br>Ana.copaescu@austin.org.au
Vanderbilt University Medical Center	1211 Medical Center Dr, Nashville, TN 37232, United States	A/Prof Elizabeth Phillips<br>Ana Copaescu	0466067000	elizabeth.j.phillips@vumc.org<br>Ana.copaescu@austin.org.au
Duke University Medical Center	10 Duke Medicine Cir, Durham, NC 27710-1000, United States	A/Prof Nicholas Turner	919-328-0148	Nick.turner@duke.edu

3. Introduction/Background Information

b. Lay Summary

Penicillin allergies are a major burden on patients and health care worldwide. Currently, up to 1 in 4 Australian patients admitted to hospital will report an antibiotic allergy, many of which limit appropriate antibiotic use and lead to poorer health outcomes. In some instances, patients will report what is considered a “low-risk” penicillin allergy and could be eligible for a penicillin challenge (i.e. a simple test dose) to determine if they are still allergic. However, the standard of care for a Penicillin allergy assessment includes skin testing (prick testing and intra-dermal testing) and, if negative, a graded oral challenge (1/10 of the dose followed by 1/1 of the dose). We have developed a penicillin allergy clinical decision rule, the PEN-FAST, that facilitates point-of-care risk assessment of patient-reported penicillin allergies. A score of less than 3 is associated with a high negative predictive value [2]. A direct oral challenge will not only reduce the time spent in clinic but also the related costs and allow an increased access to the allergy clinic for the patients with a severe allergy phenotype.

c. Introduction

Penicillin allergies are highly prevalent in the health-care setting and associated with second-line inferior antibiotics being prescribed. An incorrect penicillin allergy label leads to increased risk of resistant organisms, side effects from second-line antibiotics as well as increased medical costs [3]. Over 50% of reported penicillin allergies are considered low risk and can be identified and removed (i.e. de-labelled) via point-of-care single test dose provocation (i.e. challenge). However, the safety and efficacy of antibiotic allergy assessment, such as PEN-FAST, followed by direct oral penicillin provocation based on this validated clinical tool’s score is unknown.

This study aims to compare skin testing followed by oral drug challenge, if negative, in the outpatient allergy clinic to a direct oral penicillin provocation (i.e. test dose) when the PEN-FAST score is less than 3.

d. Background information

Patient-reported penicillin allergies result in poor health outcomes for patients and drive inappropriate antibiotic prescribing, antimicrobial resistance and healthcare costs [4-8].

Our previous work has shown that more than 85% of penicillin allergies can be removed by formal skin prick allergy testing [9], and 96-98% with low-risk allergies can be removed by point-of-care oral provocation [10]. Our group has internally and externally validated a novel penicillin allergy clinician decision rule (PEN-FAST) that is able to identify low risk penicillin allergies with a negative predictive value of 96% (95% CI 94-98%) [2].

The only currently available prospective randomized controlled trial evaluated the safety of drug challenge in a low-risk penicillin allergy patient group [3]. Specifically, the authors compared skin testing followed by drug challenge (if skin testing negative) versus direct challenge and showed that the direct challenge was a safe and effective alternative for delabeling penicillin allergy. In our international cohort, we will use the validated clinical tool, PEN-FAST, to determine if a score of less than 3 is as safe and effective as standard of care defined as skin testing followed by drug challenge if negative.

Therefore, whilst validated tools exist to enable inpatient penicillin assessment and de-labelling, limited evidence is available regarding the safety and efficacy in the outpatient clinic. The ability to deliver point-of-care penicillin allergy testing for a large cohort of patients, without skin testing, will improve patient access to testing and utilization of preferred penicillin antibiotics.

4. Study Objectives

e. Hypothesis

Using the PEN-FAST tool in an outpatient clinic setting leads to safer and faster assessments in Penicillin Allergic patients compared to standard of care (skin testing followed by oral provocation if negative).

f. Study Aims

Evaluate the non-inferiority of using PEN-FAST score guided management compared to standard care (skin testing followed by oral provocation if negative).

g. Outcome Measures

Primary outcome:

The difference in the proportion of patients with a positive oral provocation (i.e. immune-mediated reaction)

Secondary outcomes

Feasibility outcome measure:

- Proportion of patients referred to the outpatient allergy clinic that are eligible for intervention (i.e randomization) as per protocol [Eligibility to screened ratio]
- Feasibility of recruitment defined as the proportion of patients consenting to participate in the study as per protocol from eligible patients. [Recruitment to eligibility ratio].
- Feasibility of intervention delivery defined as the proportion of patients randomized to the intervention arm who had the intervention delivered as per protocol. [Intervention to recruitment ratio]

Safety outcome measures:

- Safety: The proportion of patients with a penicillin allergy who experience an antibiotic associated immune mediated adverse event OR severe adverse drug reaction as per protocol definitions.
- The proportion of patients with a penicillin allergy who experience an antibiotic associated non-immune mediated adverse event

- Protocol compliance

Exploratory efficacy outcomes
- Proportion of patient with positive Penicillin Skin Testing
- Proportion of patients with non-immune mediated positive oral challenges
- Proportion of patients with severe adverse reaction – anaphylaxis/death
- Time from randomization to delabelling
- Number of appointments required for Penicillin delabelling.
- Estimate costs (cost of skin testing versus direct drug challenge)
- Assessment with the Pre-Questionnaire and the 6 months follow-up Questionnaire

5. STUDY DESIGN

h. STUDY TYPE & DESIGN & SCHEDULE

This is an international multi-center, prospective non-inferiority randomized clinical trial (Summary in **Figure 1**) to be conducted in the outpatient services at Austin Health, Peter MacCallum Cancer Centre, Royal Melbourne Hospital, Centre hospitalier de l'Université de Montréal (CHUM), McGill University Health Centre (MUHC) and Vanderbilt University Medical Center commencing July 2020.

Eligible patients referred to the outpatient clinic reporting a penicillin allergy will be identified and assessed with a standard clinical history and the calculation of the PEN-FAST score (**Figure 2 and Appendix 2**). PEN-FAST is a three-point clinical assessment tool recently externally validated in a multicenter study, with a PEN-FAST score of < 3 associated with 96.7% negative predictive value [2]. We will include 380 patients and allocate them 1:1 ratio to the intervention group (PEN-FAST tool followed by oral penicillin provocation if score is less than 3) and control group (standard of care).

Only the patients with a score less than 3 will be randomized 1:1 following informed written consent to the intervention arm (direct oral penicillin challenge) or standard of care (skin testing following by oral challenge if negative).

Study population: Adult patients ≥ 18 years reporting a penicillin allergy

Primary objective: The difference in the proportion of patients with a positive oral provocation (i.e. immune-mediated reaction)

Pre-randomisation questionnaire:

Prior to the intervention or standard of care procedures, participants will be asked to complete a Drug Hypersensitivity Pre-Questionnaire (**Appendix 3**).

Intervention:

The patient will receive a single dose of oral penicillin, following baseline vital signs (i.e. temperature, heart rate, blood pressure, respiratory rate, skin check). If the patient reports a reaction to either penicillin unspecified, penicillin G, penicillin V, semi-synthetic anti-staphylococcal penicillins, ampicillin, amoxicillin or amoxicillin/clavulanate, he/she will be administered either the implicated drug or amoxicillin, consistent with site local practice. The dose used for amoxicillin is 250 mg or the lowest available dose according to center availability. The implicated drug should also be administered at the lowest available dose. For the amoxicillin/clavulanate allergic patients, they will retain an allergy label to clavulanate.

Nursing staff will repeat vital signs as needed after oral challenge while observing for signs of an immune mediated adverse reaction. If at any stage an antibiotic associated adverse event is noted, standard of care treatment is offered by the attending clinician (ex. adrenalin for immediate hypersensitivity reaction).

Control:

Routine management as per the treating clinicians that include skin prick and intradermal beta-lactam testing, followed by oral penicillin challenge in the setting of negative skin testing.

There will be no additional blood sampling or testing for patients in either arm of the trial. Patients in both groups will be able to directly contact a member of the clinical team (phone or email) if any serious or antibiotic associated adverse events occurs in the next 24 to 48 hours.

Follow-up Telephone Questionnaire: A 6-month post-randomisation telephone questionnaire, based on previous publications [11], will help assess the impact of outpatient delabeling on patient perceptions of their allergy status (Annexe 4).

FIGURE 1 – OVERVIEW OF THE STUDY DESIGN

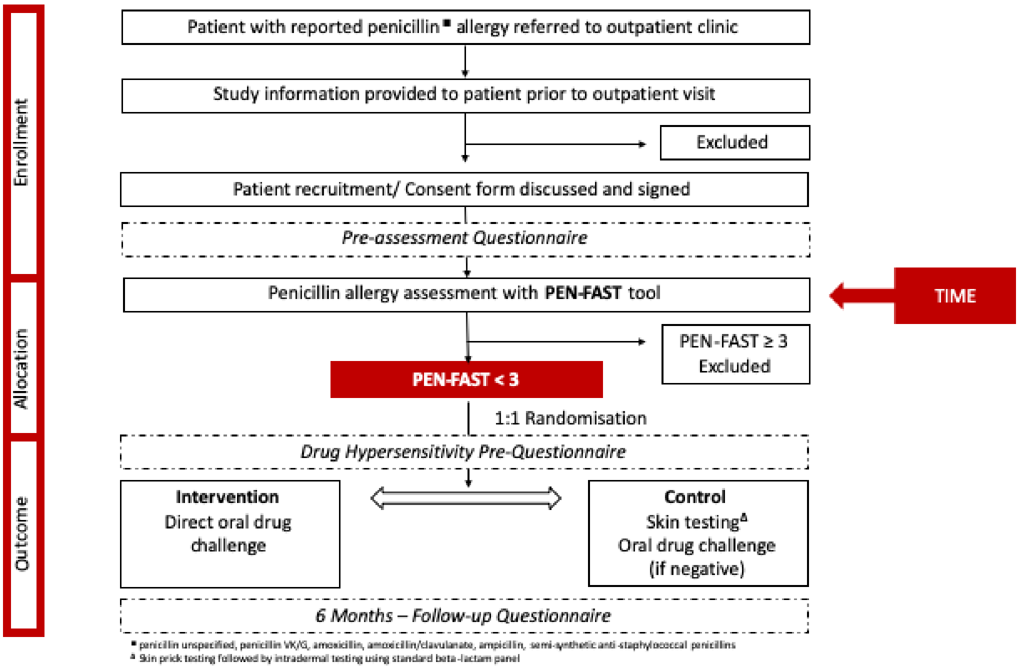

Figure 2 – PEN-FAST clinical decision rule

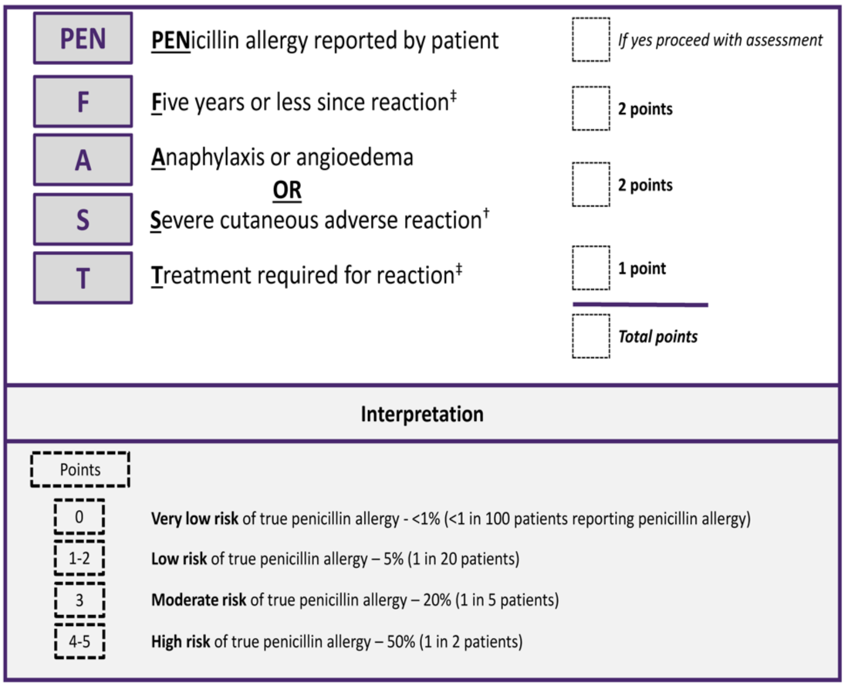

† Severe cutaneous adverse drug reaction – Stevens-Johnson syndrome (SJS), toxic epidermal necrolysis (TEN), drug reaction with eosinophilia and systemic symptoms (DRESS), acute generalized exanthematous pustulosis (AGEP)
‡ Or Unknown

i. Standard Care and Additional to Standard Care Procedures

Standard Care Procedures		
**Procedures**	**Time/Visit**	**Dosage/Volume**
Skin testing	15-20 min	n/a
Single dose Oral drug Challenge	60-120 min	Depending on the drug
Multiple dose Oral drug Challenge	Every 20-30 min and 60-90 min after the last dose	Depending on the drug

j. Randomization

Permuted block design randomisation will be used, stratified by the hospital site. Randomization will be performed via *RedCap* just prior to the intervention. The allocation sequence will be concealed until the time of the randomisation.

k. Study methodology

All eligible patients who have a history of penicillin allergy will be evaluated using PEN-FAST tool and those with PEN-FAST score <3 will be randomized to either:

Single dose Oral Drug challenge

OR

Standard of care (skin testing followed by oral drug challenge if negative)

6. Study Population

l. Recruitment Procedure

All adult patients referred to the outpatient clinic that have a documented or reported penicillin allergy will be screened for eligibility.

m. Inclusion Criteria

1. Adult patients referred to the outpatient allergy clinic for a penicillin allergy history
2. Willing and able to give consent

n. Exclusion Criteria

Patients will be **EXCLUDED** from the study if ONE of the following criteria is present:

1. Patient age is < 18 years;
2. Patients with a PEN-FAST score ≥3
3. Pregnancy
4. Any other illness that, in the investigator's judgement, will substantially increase the risk associated with subject's participation in this study
5. Patients with history of type A adverse drug reaction, drug-associated anaphylaxis, idiopathic urticaria/anaphylaxis, mastocytosis, serum sickness, blistering skin eruption or acute interstitial nephritis;
6. Patients where the allergy history was not able to be confirmed
7. Patients on concurrent antihistamine therapy
8. Patients receiving more than stress dose steroids (i.e. > 50mg QID hydrocortisone [or steroid equivalent])

o. Consent

All eligible patients will be provided with a verbal explanation of the project. They will also be provided with a paper or electronic consent form (depending on the local practices). If required, a copy will be given to the patients to further discuss with their treating medical team or family.

A thorough assessment of the participant's competence and capacity to make a valid informed decision will be made by one of the study investigators prior to the patient being recruited. All patients will be deemed competent if they:

1. Are able to comprehend and retain information relevant to making the decision;
2. Understand the information and implications of the decision;
3. Are able to weigh the information in the balance and arrive at a decision.

7. Participant Safety and Withdrawal

p. Risk Management and Safety

Several previous studies performed at Austin Health have assessed the safety of oral penicillin provocation utilizing validated risk assessment tools [2, 10, 12]. All previous studies have reported no serious adverse effects from such treatment. Several have reported potential benefit. Accordingly, we believe the study carries a high level of safety.

The types of side effects include allergic reactions such as mild rash (i.e. 2 in 100) or anaphylaxis (i.e.1 in 10,000). Anaphylaxis is a serious allergic response, typically involving

more than one body system. Symptoms such as hives, swelling and trouble breathing usually begin within 5 - 30 minutes of exposure to an allergen and may lead to anaphylactic shock which can be fatal if not treated immediately. Serious reactions such as anaphylaxis have not been reported with this test dose procedure but are theoretically possible. Other side effects such as itch, nausea, vomiting, diarrhea, abnormalities of liver or kidney tests are also possible, although very unlikely.

Because of this risk, the direct oral challenge is done in an outpatient hospital setting with surveillance from the medical staff (doctors and nurses). All the outpatient clinics are equipped with anaphylaxis managements kits and have access to resuscitation equipment as needed. Patients are supervised in the clinic for minimum one hour after the challenge.

An independent data safety management board (DSMB) will be established to review the progress of the study and monitor adherence to the protocol, participant recruitment, outcomes, complications, and other issues related to participant safety. They will also monitor the assumptions underlying sample size calculations for the study and alert the investigators if an increased recruitment effort is required. The DSMB will make recommendations as to whether the study should continue or be terminated, consider participant safety or other circumstances as grounds for early termination, including either compelling internal or external evidence of treatment differences or feasibility of addressing the study hypotheses (e.g. poor participant enrolment).

q. Handling of Withdrawals

Participants may withdraw from the study at any point. In these circumstances, the participant's data collected before the withdrawal might be included in the analysis.

r. Replacements

No withdrawals post randomization will be replaced.

8. Statistical Methods

s. Sample Size Estimation & Justification

The null hypothesis is that the difference in the proportion of positive allergy tests is not larger than 5 % (non-inferiority margin). To achieve 80% power assuming the event rate in control group being 4% and type 1 error probability of 5 % (one-sided), a total of 380 patients need to be randomized (190 per group).

t. Statistical Methods To Be Undertaken

Results will be presented according to CONSORT guidelines. Analysis will be on intention-to-treat principle with additional per-protocol analysis.

Descriptive statistics for patient characteristics, penicillin allergy history and the reasons patients did not undergo penicillin allergy assessment will be presented. Continuous variables will be presented as median (interquartile range) and categorical variables as frequency (percentage).

Primary result will be presented as the absolute difference of the primary outcome between groups with 90% confidence interval (due to one-sided 5% significance used in sample calculation). If the upper level of this interval is greater than non-inferiority margin (5%), the null hypothesis cannot be rejected. All other secondary outcomes will be presented as difference in the proportion with 95% CI and OR with 95 % CI. Continuous outcomes will be compared using t-test or rank sum test (depending on the distribution). All analyses will be conducted using STATA v16.

9. Storage of Blood and Tissue Samples

u. Details of where samples will be stored, and the type of consent for future use of samples

Not applicable.

10. Data Security & Handling

v. Details of where records will be kept & How long will they be stored

Patient clinical details and demographics will be recorded on data collections forms usually used in the outpatient clinic at each participating center. Completed forms will be kept in the Department of Infectious Diseases at the Austin hospital, Peter MacCallum Cancer Center, Royal Melbourne Hospital and the Allergy-Immunology departments at the CHUM, MUHC and Vanderbilt University Medical Center.

The collected data from every institution will then be stored on an electronic database (i.e. REDcap Austin Health) on password-protected computers. Paper data and study related documents used in this study will be re-identified and only a master log will be maintained to identify participants and their study data. The log will be locked in a protected office. All data for study will be retained for a period of fifteen years after which all electronic and paper data will be destroyed in accordance with hospital policy in place at the time. If the combination of these routinely collected data and information derived from this study provides useful clinical insights into the management of penicillin allergy, we plan to publish our findings. Authorship will be determined by the Investigational team with reference to the International Committee of Medical Journal Editors guidelines. Only aggregated non-identifiable patient data will be presented or published.

w. Confidentiality and Security

An independent data safety management board (DSMB) will be established to review the progress of the study and monitor adherence to the protocol, participant recruitment, outcomes, complications, and other issues related to participant safety.

x. Ancillary data

Not applicable.

11. APPENDIX 1 – DRUG ALLERGY TESTING CONCENTRATIONS

A. Skin Prick Testing (Read at 15 minutes)

Time applied   Time read

	Wheal	Flare	Positive	Negative	Signature
Histamine 10mg/ml					
Sodium Chloride 0.9%					
Diater PPL (major determinant)*					
Diater MDM (minor determinant) (if available)					
Ampicillin 25mg/ml					
Penicillin 10 000 U/ml					

* Depending on site availability, major determinant Pre-Pen® can also be used

B. Intradermal Testing (0.02 ml)

Time applied   Time read

	Baseline reading	Final reading	Result	Signature
Sodium Chloride 0.9%				
Diater PPL (major determinant)				
Diater MDM (minor determinant) (if available)				
Ampicillin 25mg/ml				
Penicillin 10 000 U/ml				

12. APPENDIX 2 – PEN-FAST CLINICAL DECISION RULE

PALACE Study

The use of penicillin allergy clinical decision rule to enable direct oral penicillin provocation – A multicenter randomized control trial

**PEN**	**PEN**icillin allergy reported by patient	*If yes proceed with assessment*
**F**	**F**ive years or less since reaction‡	**2 points**
**A**	**A**naphylaxis or angioedema **OR**	**2 points**
**S**	**S**evere cutaneous adverse reaction†	
**T**	**T**reatment required for reaction‡	**1 point**
		Total points

Interpretation

Points	
0	**Very low risk** of true penicillin allergy - <1% (<1 in 100 patients reporting penicillin allergy)
1-2	**Low risk** of true penicillin allergy – 5% (1 in 20 patients)
3	**Moderate risk** of true penicillin allergy – 20% (1 in 5 patients)
4-5	**High risk** of true penicillin allergy – 50% (1 in 2 patients)

† Severe cutaneous adverse drug reaction – Stevens-Johnson syndrome (SJS), toxic epidermal necrolysis (TEN), drug reaction with eosinophilia and systemic symptoms (DRESS), acute generalized exanthematous pustulosis (AGEP)
‡ Or Unknown

13. Appendix 3 – Drug Hypersensitivity Pre-Questionnaire

**Please answer yes, no or non-applicable (N/A) to the following questions**. M**ark the appropriate box with an x.**	**Yes**	**No**	**N/A**
1. Would you like the allergist's/infectious disease's physician opinion before taking medications prescribed by other specialists?	□	□	□
2. Do you talk to others about your allergy problem?	□	□	□
3. Is your family aware of your problem?	□	□	□
4. Is your partner conscious of your problem?	□	□	□
5. Is your family doctor aware of your drug allergy problem?	□	□	□
6. Is your community pharmacist aware of your drug allergy problem?	□	□	□
6. Would you be happy to have penicillin again in the community after a negative test result in clinic?	□	□	□

The following questions concern the influence your drug allergy has on your quality of life. Answer every question by marking the appropriate box with an x. You may choose from one of the following answers.

**0**	**1**	**2**	**3**	**4**
**Not at all**	**Slightly**	**Moderately**	**Very**	**Extremely**

	**0**	**1**	**2**	**3**	**4**
6. Do you feel different from others?	□	□	□	□	□
7. Do you feel unluckier from others?	□	□	□	□	□
8. Is it that even a little discomfort is a problem for you?	□	□	□	□	□
9. Is your job efficiency affected by the problem of your allergy to medications?	□	□	□	□	□
10. Do you feel helpless?	□	□	□	□	□
11. Do you sleep badly?	□	□	□	□	□
12. Do you feel embarrassed in relationships with others?	□	□	□	□	□
13. Since you are unable to take medications, does every illness limit you more than other people?	□	□	□	□	□
14. Do you have difficulties concentrating?	□	□	□	□	□
15. Does your allergy problem interfere with your sexual life?	□	□	□	□	□
16. Do you feel anguished due to your problem of allergy reaction?	□	□	□	□	□

17. Do you feel ill?	☐	☐	☐	☐	☐
18. Are you restricted in your nutrition from fear of substances you might be allergic to?	☐	☐	☐	☐	☐
19. Are you afraid of being administered a medication during an emergency to which you are allergic?	☐	☐	☐	☐	☐
20. Do you feel you can't cope with your allergy problem?	☐	☐	☐	☐	☐
21. For each disease, would you be confident that there is a medication that you can safely take?	☐	☐	☐	☐	☐
22. Are you afraid you could not deal with the pain?	☐	☐	☐	☐	☐
23. Do you feel anxious due to your problem of allergy reaction?	☐	☐	☐	☐	☐
24. Does your problem influence your relationships with other people?	☐	☐	☐	☐	☐
25. Are you in a bad mood due to your problem of allergy reaction?	☐	☐	☐	☐	☐
26. Do you feel frightened due to your problem of allergy reaction?	☐	☐	☐	☐	☐
27. Do you worry every time you take a medication different from ones that cause your allergic reactions?	☐	☐	☐	☐	☐
28. Do you feel tired during the day because you sleep badly at night?	☐	☐	☐	☐	☐
29. Do you give up leisure activities (sport, vacations, trips) because of your problem?	☐	☐	☐	☐	☐
30. Does the idea of taking a medicine make you feel anxious?	☐	☐	☐	☐	☐
31. Are you annoyed by frequent medical controls?	☐	☐	☐	☐	☐
33. Does the problem of adverse reaction to medications affect your life?	☐	☐	☐	☐	☐
34. How likely are you to believe a negative penicillin allergy test result	☐	☐	☐	☐	☐
35. How likely do you think it is that your penicillin allergy test will be negative	☐	☐	☐	☐	☐

14. Appendix 4 – 6 Months follow-up telephone questionnaire - Telephone survey script [11]

Telephone survey script

Verbal consent script for patients who were randomized in the trial.

"Hello could I please speak to (patient's full given name and surname)?"

Hello, I am _______, (name and function in the hospital). You have participated in a study on Penicillin allergy, the PALACE Study, about 6 months ago. We are now contacting for the second part of the study in order to find out what antibiotics you have used after antibiotic allergy testing at our center (Name the center). You have been selected to be involved in this project because you came to our center and had your antibiotic allergy reviewed.

Before we proceed further, can I please confirm your full name and date of birth?

If you agree to continue to participate in this study, we will ask you some questions about your allergies and what antibiotics you have taken and any problems with your antibiotics recently. Usually the interview takes about 10 minutes, but if we identify some problems with your allergies and we help you to solve these problems, it might take longer. If we identify some problems, we might ask for your permission to contact your local doctor or the antibiotic allergy service at our center that can help you to solve these problems. Taking part in this interview is completely voluntary and will not affect your future care at our center.

If the patient is not at home:

"Is there a time that I could call back to speak with (patient's name)?"

If the patient is busy:

"Is there another time that I could call back that would be convenient?"

Patient questions

Do you consent for us to check what antibiotics you have been prescribed by your doctors in the community and dispensed by your pharmacy? We can check this via a program called (name the local program depending on the center).

1. Do you remember having a test dose of penicillin in the outpatient clinic?
a. **If Yes**, please tell me whether you agree with these statements:
i. "I felt safe during the test dose."
1. Strongly agree
2. Agree
3. Neutral
4. Disagree
5. Strongly disagree

ii. "I recommend the penicillin assessment to other patients with a penicillin allergy."
1. Strongly agree
2. Agree
3. Neutral

4. Disagree
5. Strongly disagree

iii. What was the result of your penicillin assessment in the clinic?
1. Penicillin allergy removed
2. Penicillin allergy confirmed
3. I don't know

iv. Did you have any late reaction to assessment after the observation period?
1. If Yes, state reaction:
2. What treatment was required? (eg, General Practitioner visit, antihistamines, topical steroids, readmission to hospital)

v. Have you received an antibiotic since the test?
1. If yes, what was the name of the antibiotic?
2. If unable to recall, prompt: Was a "penicillin"?
3. If yes (ie, penicillin received), did you have any reaction to the penicillin?

vi. Did you receive a letter about your allergy post-testing? Y/N

vii. Do you feel you know more about penicillin allergies? Y/N

viii. Do you feel you know more about your reactions to penicillin? Y/N

ix. Are you still avoiding penicillin(s)?

1. If Yes, please explain why? Free-text (Investigator to categorize later)

x. Did you consider yourself allergic to penicillin? Y/N

1. If Yes, the next time you are admitted to hospital, would you say that you are allergic to penicillin?

b. Do you have any comments about the testing, either good or bad, that you would like to pass on to the team? [freetext]

If the patient states that they are still avoiding penicillin (x) or they consider themselves allergic to penicillin (xi) and you have assessed them to be able to participate in a qualitative interview,

then say:

"We would like to explore these issues further. This would involve another phone interview. Would you be interested in participating? What would be a good time to call you?"

End—"That is the end of the questions. Thank you very much for your time."

15. REFERENCES

1. Rawlins, M. and J. Thompson, *Textbook of adverse drug reactions*. 1977, Oxford: Oxford University Press.
2. Trubiano, J.A., et al., *Development and Validation of a Penicillin Allergy Clinical Decision Rule.* JAMA Intern Med, 2020.
3. Mustafa, S.S., K. Conn, and A. Ramsey, *Comparing Direct Challenge to Penicillin Skin Testing for the Outpatient Evaluation of Penicillin Allergy: A Randomized Controlled Trial.* J Allergy Clin Immunol Pract, 2019. **7**(7): p. 2163-2170.
4. Trubiano, J.A., et al., *Antimicrobial allergy 'labels' drive inappropriate antimicrobial prescribing: lessons for stewardship.* J Antimicrob Chemother, 2016. **71**(6): p. 1715-22.
5. MacFadden, D.R., et al., *Impact of Reported Beta-Lactam Allergy on Inpatient Outcomes: A Multicenter Prospective Cohort Study.* Clin Infect Dis, 2016. **63**(7): p. 904-910.
6. Trubiano, J.A., et al., *Old but not forgotten: Antibiotic allergies in General Medicine (the AGM Study).* Med J Aust, 2016. **204**(7): p. 273.
7. Blumenthal, K.G., et al., *Risk of meticillin resistant Staphylococcus aureus and Clostridium difficile in patients with a documented penicillin allergy: population based matched cohort study.* BMJ, 2018. **361**: p. k2400.
8. Moran, R., et al., *Antibiotic allergy labels in hospitalized and critically ill adults: A review of current impacts of inaccurate labelling.* Br J Clin Pharmacol, 2019. **85**(3): p. 492-500.
9. Trubiano, J.A., et al., *Impact of an Integrated Antibiotic Allergy Testing Program on Antimicrobial Stewardship: A Multicenter Evaluation.* Clin Infect Dis, 2017. **65**(1): p. 166-174.
10. Trubiano, J.A., et al., *The Safety and Efficacy of an Oral Penicillin Challenge Program in Cancer Patients: A Multicenter Pilot Study.* Open Forum Infect Dis, 2018. **5**(12): p. ofy306.
11. Wilson, A., J.A. Trubiano, and K.Y.L. Chua, *Patient perspectives on antibiotic allergy delabeling: Enablers and barriers.* J Allergy Clin Immunol Pract, 2020. **8**(10): p. 3637-3639 e5.
12. Devchand, M., et al., *Pathways to improved antibiotic allergy and antimicrobial stewardship practice: The validation of a beta-lactam antibiotic allergy assessment tool.* J Allergy Clin Immunol Pract, 2019. **7**(3): p. 1063-1065 e5.
