# Supplementary material for: Risk of Self-Reported Penicillin Allergy Despite Removal of Penicillin Allergy Label: Secondary Analysis of the PALACE Randomized Clinical Trial
Source: JAMA Netw Open. 2024 Aug 15;7(8):e2429621. doi: 10.1001/jamanetworkopen.2024.29621 (PMC11327879; doi:10.1001/jamanetworkopen.2024.29621)
Supplement: Supplement 2. — Statistical Analysis Plan [file jamanetwopen-e2429621-s002.pdf]

PALACE Study

The use of penicillin allergy clinical decision rule to enable direct oral penicillin provocation – A multicenter randomized control trial – PALACE Study

Statistical Analysis Plan

Version: 2.0 (Final)

Date: 10/12/2022

Authors:
Sara Vogrin, The Department of Medicine, University of Melbourne
Dr Ana Copaescu, McGill University Health Centre/Research Institute of the McGill University Health Centre
Fiona James, Centre for Antibiotic Allergy and Research, Department of Infectious Diseases, Austin Health
A Prof Jason Trubiano, Centre for Antibiotic Allergy and Research, Department of Infectious Diseases, Austin Health

Study identifiers:
ClinicalTrials.gov Identifier: NCT04454229

Centre for Antibiotic Allergy and Research
Optimising antibiotic therapy

Austin HEALTH

TABLE OF CONTENTS

2
1 ADMINISTRATIVE INFORMATION 3
1.1 STUDY IDENTIFIERS 3
1.2 REVISION HISTORY 3
1.3 CONTRIBUTORS TO THE STATISTICAL ANALYSIS PLAN 3
2 STUDY SYNOPSIS 5
2.1 PATIENT POPULATION 6
2.2 OUTCOMES 6
2.3 INTERVENTION 7
2.4 RANDOMISATION AND BLINDING 7
2.5 SAMPLE SIZE 7
3 STATISTICAL ANALYSIS 8
3.1 GENERAL PRINCIPLES 8
3.2 INTERIM ANALYSES 8
3.3 MULTIPLICITY ADJUSTMENT 8
3.4 DATA SETS TO BE ANALYSED 8
3.5 SUBJECT DISPOSITION 8
3.6 PATIENT CHARACTERISTICS AND BASELINE COMPARISONS 8
3.7 COMPLIANCE TO STUDY INTERVENTION(S) 9
3.8 ANALYSIS OF THE PRIMARY OUTCOME 9
3.9 ANALYSIS OF SECONDARY OUTCOMES 10
4 REFERENCES 11

1 ADMINISTRATIVE INFORMATION

1.1 STUDY IDENTIFIERS

- Protocol: version 4, date 09/03/2021
- ClinicalTrials.gov register Identifier: NCT04454229

1.2 REVISION HISTORY

Version	Date	Details
0.1 (draft)	28/10/2022	First draft by Sara
1.0 (final)	6/11/2022	Updated following review by co-authors
2.0	10/12/2022	Updated the definition of primary outcome which matches ethics approved protocol (misinterpretation in the previous version)

1.3 CONTRIBUTORS TO THE STATISTICAL ANALYSIS PLAN

1.3.1 ROLES AND RESPONSIBILITIES

Names	Affiliation	Role on study	SAP contribution
Sara Vogrin	The Department of Medicine, University of Melbourne	Study statistician	Prepared initial draft and revisions
Fiona James	Centre for Antibiotic Allergy and Research, Department of Infectious Diseases, Austin Health	Project manager	Reviewed drafts
Dr Ana Copaescu	McGill University Health Centre/Research Institute of the McGill University Health Centre	Chief investigator	Reviewed drafts
A/Prof Jason Trubiano	Centre for Antibiotic Allergy and Research, Department of Infectious Diseases, Austin Health	Chief investigator	Reviewed drafts

1.3.2 APPROVALS

The undersigned have reviewed this plan and approved it as final. They find it to be consistent with the requirements of the protocol as it applies to their respective areas. They also find it to comply with ICH-E9 principles and, in particular, confirm that this analysis plan was developed in a completely blinded manner (i.e. without knowledge of the effect of the intervention(s) being assessed).

Sara Vogrin 10/12/2022

Dr Ana Copaescu 10/12/2022

Fiona James 11/12/2022

A/Prof Jason Trubiano 10/12/2022 Type text here

2 STUDY SYNOPSIS

PALACE is a prospective, international, parallel, two-arm, non-inferiority, multicenter randomized control trial evaluating the safety of direct oral challenge vs skin testing prior to oral challenge in adult patients with low-risk penicillin allergy (identified as PEN-FAST <3).[1]

The study will test the hypothesis that in an adult population (>18 years) with low-risk penicillin allergy (defined as PEN-FAST score <3), receiving direct oral challenge will be non-inferior to receiving skin testing prior to oral challenge. Non-inferiority will be announced if one sided 95% confidence interval of the risk difference of a positive oral challenge (an immune-mediated reaction) does not exceed 5%.

Given the design of the study, post-randomization events are expected to be minimal – exclusion post-randomization due to ineligibility will be treated using a principal stratum strategy, while all other post-randomization events will be treated using treatment policy strategy.

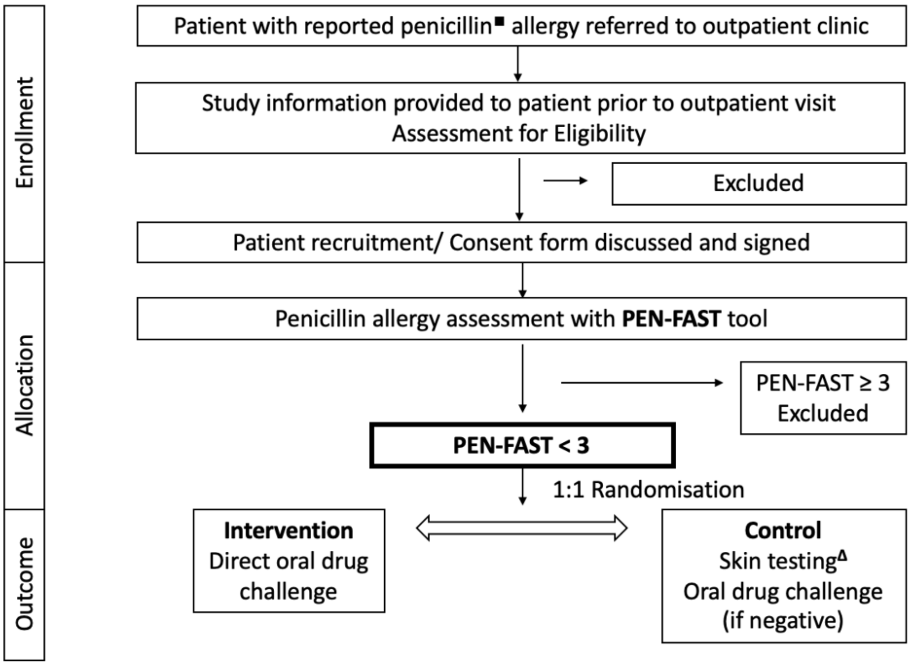

■ penicillin unspecified, penicillin VK/G, amoxicillin, ampicillin, dicloxacillin, flucloxacillin
Δ Skin prick testing followed by intradermal testing using standard beta-lactam panel (**Table 1**)

2.1 PATIENT POPULATION

The study will be conducted in 6 outpatient clinics in major tertiary centers in Australia (3 sites), Canada (1 site) and US (2 sites).

2.1.1 INCLUSION CRITERIA

- Adult patients referred to the outpatient allergy clinic for a penicillin allergy history.
- Willing and able to give consent.

2.1.2 EXCLUSION CRITERIA

- Age < 18 years.
- PEN-FAST score ≥3.
- Pregnancy.
- Any other illness that, in the investigator's judgment, will substantially increase the risk associated with the subject's participation in this study.
- Patients with a history of type A adverse drug reaction, drug-associated anaphylaxis, idiopathic urticaria/anaphylaxis, mastocytosis, serum sickness, blistering skin eruption or acute interstitial nephritis.
- Patients where the allergy history was not able to be confirmed.
- Patients on concurrent antihistamine therapy.
- Patients receiving more than stress dose steroids (i.e. >50mg QID hydrocortisone [or steroid equivalent]).

2.2 OUTCOMES

2.2.1 PRIMARY OUTCOME

- The difference in the proportion of patients with a positive oral provocation (an immune-mediated reaction) between the intervention and control group.

2.2.2 SECONDARY OUTCOMES

- **Feasibility outcomes:**
- Proportion of patients referred to the outpatient allergy clinic that are eligible for intervention (i.e randomization) as per protocol [Eligibility to screened ratio]
- Feasibility of recruitment defined as the proportion of patients consenting to participate in the study as per protocol from eligible patients. [Recruitment to eligibility ratio].
- Feasibility of intervention delivery defined as the proportion of patients randomized to the intervention arm who had the intervention delivered as per protocol. [Intervention to recruitment ratio]

- **Safety outcomes:**
- The proportion of patients with a penicillin allergy who experience an antibiotic associated immune mediated adverse event OR severe adverse drug reaction as per protocol definitions within each arm and their difference.
- The proportion of patients with a penicillin allergy who experience an antibiotic associated non-immune mediated adverse event within each arm and their difference.
- Protocol compliance within each arm.

- **Efficacy outcomes:**
- Proportion of patients with positive penicillin skin testing within the control arm.
- Proportion of patients with non-immune mediated positive oral challenges within each arm and their difference.
- Proportion of patients with severe adverse reaction within each arm and their difference (serious adverse event is defined as any adverse drug event/experience occurring at any dose that in the opinion of the investigator is causal for any of the following outcomes: death, life-threatening reaction, inpatient hospitalization, persistent or significant disability/incapacity, congenital anomaly/birth defect or requires intervention to prevent permanent impairment or damage).
- Time from randomization to delabelling within each arm and their difference.
- Number of appointments required for penicillin delabelling within each arm and their difference.
- Estimate costs of testing within each arm and their difference.
- Qualitative assessment of the Pre-Questionnaire and 6-month Follow-Up Questionnaire

2.3 INTERVENTION

Intervention: A single dose of oral penicillin, following baseline vital signs (heart rate, blood pressure, respiratory rate).

Control: Routine management as per the treating clinicians that include skin prick and intradermal beta-lactam testing, followed by oral penicillin challenge in the setting of negative skin testing. Vital signs recorded as per intervention group.

2.4 RANDOMISATION AND BLINDING

Permuted block design randomization will be used, stratified by the clinical site. Randomization will be performed via REDCap (Research Electronic Data Capture hosted at Austin Health)[2] just prior to the intervention. The allocation sequence will be concealed until the time of the randomization. Due to the nature of the control treatment (skin testing) it is impossible to blind study participants, treating clinicians or data collectors. Study investigators (unless they are treating clinicians) will be blinded.

2.5 SAMPLE SIZE

The null hypothesis is that the difference in the proportion of positive allergy tests (oral challenge or skin testing) between the intervention and control arm is not larger than 5 % (non-inferiority margin). To achieve 80% power, assuming the event rate in the control group is 4% and type 1 error probability of 5 % (one-sided), a total of 380 participants are required (190 per group). If the control group has a lower prevalence of the outcome (2.5% or 2.0%), the power of the study will remain at least 80% if up to 4.5% of the intervention group has the outcome (table below). Due to the randomization, intervention and primary outcome being collected within the same visit, loss to follow-up is expected to be minimal.

P in control	P in intervention	Power
0.045	0.045	94%
0.025	0.035	94%
0.025	0.045	84%
0.020	0.035	91%
0.020	0.045	79%

3 STATISTICAL ANALYSIS

3.1 GENERAL PRINCIPLES

While the protocol states that risk differences will be used for analysis of primary and secondary outcome, risk ratios will also be reported. Additional subgroup analysis based on participant age (<65 vs ≥65), sex and reported antibiotic was added. This analysis plan is applicable for all outcomes except cost analysis and 6-month outcomes.

Results will be reported following extension of the CONSORT 2010 guidelines for reporting of noninferiority and equivalence randomized trials[3].

Analysis will be conducted primarily using Stata (version 16.1 or above).[4]

3.2 INTERIM ANALYSES

No interim analysis will be performed. Pooled safety outcomes will be presented at regular DSMB meetings.

3.3 MULTIPLICITY ADJUSTMENT

The study has a single primary outcome and its analysis will be unadjusted for multiplicity. One-sided confidence intervals will be used to assess non-inferiority. All secondary outcomes will use two-sided 95% confidence intervals and two-sided tests with a nominal alpha level of 5% if applicable. As most of the secondary outcomes are descriptive and presented for the overall study or within a single arm, no adjustment for multiplicity will be performed.

3.4 DATA SETS TO BE ANALYSED

All analyses will be conducted on an intention-to-treat (ITT) basis. ITT population includes all patients randomized regardless of whether they received the intervention. Any post-randomization exclusions due to ineligibility (expected to be none or minimal) will be excluded from the analysis.

Sensitivity analysis will exclude patients who did not receive intervention as per protocol definitions. Additional sensitivity analysis will include adjustments for the clinical site. This will only be performed for the primary outcome.

3.5 SUBJECT DISPOSITION

Subject disposition will be presented using a CONSORT diagram[5]. This will include the number of patients that were screened, eligible, recruited, randomized and analyzed. Any exclusions will be listed and will include reasons.

3.6 PATIENT CHARACTERISTICS AND BASELINE COMPARISONS

Baseline characteristics will be presented overall and by the treatment group. Continuous variables will be summarized using the median and interquartile range, while categorical variables will be presented using frequencies and percentages.

3.7 COMPLIANCE TO STUDY INTERVENTION(S)

The details of the intervention received will be presented in a table (N and % of patients receiving each type of skin testing and type of oral challenge). The time required to complete the intervention is listed as a secondary outcome.

3.8 ANALYSIS OF THE PRIMARY OUTCOME

Primary analysis will be conducted without missing data imputation, as it is expected that missing data will be minimal. In the event missing data is present in >5% of patients, missing data will be imputed using multiple imputation and imputed data will be used for primary analysis.

3.8.1 MAIN ANALYSIS

Primary endpoint is the proportion of participants experiencing positive oral challenge (immune mediated reaction). Participants with a positive skin test will not undergo oral challenge, therefore will be counted as not achieving primary outcome. Primary analysis will be using generalized linear model (GLM) with binomial family and identity link to estimate the risk difference between intervention and control (reference will be control). This will be presented using a two-sided 90% confidence interval to represent a single-sided 95% confidence interval. If the upper limit of the confidence interval does not cross the non-inferiority margin of 5%, the study will be determined to be non-inferior.

Although clinical site was used as a stratification factor in randomization, adjusting for the site will likely cause convergence issues or produce unstable estimates given the expected small number of outcomes (5% or less) and relatively small number of centres (n=6). Therefore, the main analysis will not adjust for clinical site[6].

Results will also be presented using risk ratios with two-sided 90% confidence intervals (to represent one-sided 95% confidence intervals), which will be analyzed in the same manner, but using log link. If issues with the model convergence and imputation occur, then GLM with Gaussian family, robust standard errors and identity/log link will be used for risk difference/risk ratio respectively.

3.8.2 SUBGROUP ANALYSES

The following prespecified subgroup analysis will be performed irrespective of the outcome of primary analysis:

1. Clinical immunophenotypes (immediate vs delayed reaction)
2. Number of reported drug allergies (multiple vs single)
3. Immunocompetency (immunosuppressed vs immunocompetent)
4. Age (<65 vs ≥65)
5. Sex (male vs female)
6. Penicillin type (penicillin unspecified vs penicillin VK/G or dicloxacillin or flucloxacillin vs amoxicillin or ampicillin or amoxicillin/clavulanate)
7. Dose of oral challenge drug (250 mg vs 500 mg)
8. Type of skin testing (minor vs major vs Pre-Pen)

Subgroup analysis will be performed by including the subgroup variable and interaction with the

intervention to the GLM model (for subgroups 1-5, no interaction for 6 and 7). The number of events and percentage will be presented for each subgroup, as well as risk difference and risk ratios with 95% confidence intervals. The latter will be presented in forest plot, including p value for interaction.

3.8.3 TREATMENT OF MISSING DATA

Missing data is expected to be minimal and if present in <5%, no missing data imputation will be performed. If the missing data will be present in >5%, then multiple imputation stratified by randomization group will be performed using logistic regression model with at least 50 data sets imputed[7]. The results will be combined to obtain a pooled risk difference and risk ratio.

3.8.4 OTHER SENSITIVITY ANALYSES

Sensitivity analysis will include adjustment for clinical site and per-protocol analysis.

3.9 ANALYSIS OF SECONDARY OUTCOMES

3.9.1 FEASIBILITY OUTCOMES

All feasibility outcomes will be presented using percentage with exact two-sided 95% confidence intervals.

3.9.2 SAFETY and EFFICACY OUTCOMES

All safety and binary efficacy outcomes will be presented as counts (percentage) within each arm and comparison between arms will be same as for the main outcome (using GLM model to evaluate risk difference and risk ratio, both presented with two-sided 95% confidence intervals).

Continuous efficacy outcome (time from randomization to delabelling) will be presented as mean (SD) or median (IQR). Linear regression will be used for comparison between arms. Results will be presented as mean difference with 95% confidence intervals. Homoscedasticity of residuals will be inspected visually and if violated then outcome will be transformed using natural logarithm.

Count outcomes (number of appointments required) will be analyzed using negative binomial regression with outcome expressed as incidence rate ratio with 2-sided 95% confidence intervals. If the variability in the number of appointments required will be minimal, the outcome will be transformed into a binary variable (1 appointment vs multiple appointments) and analysis will be performed using GLM, results reported as risk difference and risk ratio with two-sided 95% confidence intervals.

4 REFERENCES

1. Copaescu AM, James F, Vogrin S, et al. Use of a penicillin allergy clinical decision rule to enable direct oral penicillin provocation: an international multicentre randomised control trial in an adult population (PALACE): study protocol. *BMJ Open* 2022; **12**(8): e063784.
2. Harris PA, Taylor R, Thielke R, Payne J, Gonzalez N, Conde JG. Research electronic data capture (REDCap)--a metadata-driven methodology and workflow process for providing translational research informatics support. *J Biomed Inform* 2009; **42**(2): 377-81.
3. Piaggio G, Elbourne DR, Pocock SJ, Evans SJ, Altman DG, Group C. Reporting of noninferiority and equivalence randomized trials: extension of the CONSORT 2010 statement. *JAMA* 2012; **308**(24): 2594-604.
4. Stata Statistical Software. Release 17 ed. College Station, TX: StataCorp LLC; 2021.
5. Moher D, Schulz KF, Altman D, Group C. The CONSORT statement: revised recommendations for improving the quality of reports of parallel-group randomized trials. *JAMA* 2001; **285**(15): 1987-91.
6. Kahan BC. Accounting for centre-effects in multicentre trials with a binary outcome - when, why, and how? *BMC Med Res Methodol* 2014; **14**: 20.
7. Sullivan TR, White IR, Salter AB, Ryan P, Lee KJ. Should multiple imputation be the method of choice for handling missing data in randomized trials? *Stat Methods Med Res* 2018; **27**(9): 2610-26.
