# Supplementary material for: Risk of Self-Reported Penicillin Allergy Despite Removal of Penicillin Allergy Label: Secondary Analysis of the PALACE Randomized Clinical Trial
Source: JAMA Netw Open. 2024 Aug 15;7(8):e2429621. doi: 10.1001/jamanetworkopen.2024.29621 (PMC11327879; doi:10.1001/jamanetworkopen.2024.29621)
Supplement: Supplement 3. — eMethods. [file jamanetwopen-e2429621-s003.pdf]

Supplemental Online Content

Copaescu AM, Vogrin S, Douglas A, et al. Risk of self-reported penicillin allergy despite removal of penicillin allergy label during the PALACE study: secondary analysis of a randomized clinical trial. *JAMA Netw Open.* 2024;7(8):e2429621.
doi:10.1001/jamanetworkopen.2024.29621

eMethods

This supplemental material has been provided by the authors to give readers additional information about their work.

© 2024 Copaescu AM et al. *JAMA Network Open.*

eMethods

The PALACE study was a multicentre, parallel, two-arm, non-inferiority, international, open-label, randomized controlled trial conducted in outpatient clinics at six centres, three in North America (United States and Canada) and three in Australia. The trial was conducted using the CONSORT reporting guidelines (ref: *Schulz KF, Altman DG, Moher D, for the CONSORT Group. CONSORT 2010 Statement: updated guidelines for reporting parallel group randomised trials*).

The PALACE study determined that direct oral penicillin challenge [DOC] (intervention arm) was non-inferior to the standard of care of penicillin skin testing followed by an oral challenge (control arm) (RD 0.0084 [90.0% CI, -1.22, 1.24], below the pre-defined non-inferiority margin 5.0%).

The protocol of the PALACE study was approved by an institutional review board at Austin Health and subsequently by the independent institutional review board at each site.

All participants provided written informed consent. Site investigators confirmed eligibility before a participant underwent randomization.

The investigator observed the participant's race and/or this was self-reported by the patient during clinical history.

While data on AEs is presented, the main focus are the AEs occurring after the initial 5-day period.

© 2024 Copaescu AM et al. *JAMA Network Open.*
