# Supplementary material for: Risk of Self-Reported Penicillin Allergy Despite Removal of Penicillin Allergy Label: Secondary Analysis of the PALACE Randomized Clinical Trial
Source: JAMA Netw Open. 2024 Aug 15;7(8):e2429621. doi: 10.1001/jamanetworkopen.2024.29621 (PMC11327879; doi:10.1001/jamanetworkopen.2024.29621)
Supplement: Supplement 4. — Data Sharing Statement [file jamanetwopen-e2429621-s004.pdf]

Data Sharing Statement

Data
Data available: No

Additional Information
Explanation for why data not available: The authors confirm that the results supporting the findings of this study are available within the article [and/or] its supplementary materials. Other data supporting the findings of this study are available from the corresponding author on request.
